# Association between nasal septal deviation and tinnitus: Insights from a 9-year nationwide cohort study

**DOI:** 10.1097/MD.0000000000040208

**Published:** 2024-10-18

**Authors:** Cha Dong Yeo, Sang Woo Yeom, Eun Jung Lee, Jong Seung Kim

**Affiliations:** a Department of Otorhinolaryngology-Head and Neck Surgery, College of Medicine, Jeonbuk National University, Jeonju, Republic of Korea; b Research Institute of Clinical Medicine of Jeonbuk National University-Biomedical Research Institute, Jeonbuk National University Hospital, Jeonju, Republic of Korea; c Department of Medical Informatics, College of Medicine, Jeonbuk National University, Jeonju, Republic of Korea.

**Keywords:** cohort study, National Health Insurance Service, septal deviation, septoplasty, tinnitus

## Abstract

Tinnitus is a condition where sound is perceived in the ear or head when no external sound stimulation is present. To date, no study has explored the correlation between nasal septal deviation (SD) and tinnitus using large-scale real-world data. This study hypothesized a potential relationship between tinnitus and SD, which we investigated using a 9-year large-scale cohort study. Nationwide cohort observational study. The SD group was selected from 1 million individuals randomly extracted from the National Health Insurance Service database. The non-SD group was obtained through propensity score matching considering several variables. The primary endpoint was tinnitus diagnosis. The study (SD) group included 10,790 individuals, and the non-SD group (control group) included 21,580 individuals. The overall hazard ratio (HR) for tinnitus in the SD group was 1.74 (95% CI: 1.62–1.89). In the subgroup analysis, the HR was 0.73 (95% CI: 0.68–0.79) for tinnitus in the male group, 1.12 (95% CI: 1.04–1.21) in the group with high economic status, 0.81 (95% CI: 0.75–0.89) in the group living in metropolitan areas, and 0.45 (95% CI: 0.42–0.49) in the younger age group (<50 years). In the SD group, the HR for tinnitus after septoplasty significantly decreased to 0.75 (95% CI: 0.63–0.90). From long-term follow-up, the prevalence of tinnitus was 1.74 times higher in the SD group compared with the control group. This phenomenon significantly decreased after septoplasty.

## 1. Introduction

Tinnitus is the perception of sound in the ear or head without any external auditory stimulus. Tinnitus can be classified as acute (lasting <6 months) or chronic (lasting >6 months). It can also be categorized into primary tinnitus, where no identifiable cause other than hearing loss is found, or secondary tinnitus, which is associated with a specific underlying cause, such as middle ear pathology or vascular disorders. Furthermore, tinnitus can be objective (where an external observer can detect the sound) or subjective (where only the patient perceives the sound), with the latter being far more common. This sound is typically described as a ringing, buzzing, or hissing noise, and it can vary in pitch and loudness. While tinnitus can be transient and benign, in some cases, it can become chronic and significantly impact a patient’s quality of life, leading to sleep disturbances, concentration difficulties, and even anxiety or depression.^[1]^ Furthermore, it has been established that there is a significant correlation between tinnitus severity and psychiatric symptoms, particularly depression and anxiety. Previous investigations have demonstrated that patients with more severe tinnitus tend to have higher depression and anxiety scores, highlighting the importance of considering mental health in tinnitus management. It is known that about 94% of people experience tinnitus at levels below 20 dB in a fully soundproofed room,^[2]^ yet this is not clinically classified as tinnitus unless it is sufficiently loud to cause discomfort or distress to the patient.^[3]^ The subjective nature of tinnitus, which relies heavily on patient self-reporting, contributes to the wide variation in its reported prevalence, which ranges from 5.2% to 42.7% depending on the definition used and the population studied.^[4–6]^

Nasal septal deviation (SD) is a structural abnormality where the nasal septum, which divides the nasal cavity, is displaced to one side. This condition can lead to several complications, including chronic nasal and paranasal sinus inflammation, recurrent sinus infections, and obstruction of the sinuses, resulting in difficulty breathing through the nose.^[7]^ The most common causes of SD include trauma to the nose, either during birth or from injury later in life, as well as congenital conditions and growth abnormalities of the nasal cartilage. SD is highly prevalent, with an incidence rate of approximately 68% worldwide, and a reported prevalence of 51.1% among Koreans over the age of 12 in 2011.^[8]^ Despite its commonality, SD is often overlooked unless it causes significant symptoms.

Recent research has suggested that there may be a link between SD and various ear-related symptoms, including tinnitus. The potential mechanisms underlying this association include altered airflow dynamics, Eustachian tube dysfunction, and increased pressure in the middle ear, all of which could potentially contribute to the perception of tinnitus. However, to date, no study has systematically explored the correlation between SD and tinnitus using large-scale, real-world data. Understanding this relationship could have significant implications for the management of patients with SD, particularly those who suffer from chronic tinnitus. This study aims to evaluate the quantitative relationship between SD and tinnitus, leveraging a large, nationwide cohort to provide robust evidence on this potential association.

## 2. Methods

This study used the National Health Insurance Service–National Sample Cohort (NHIS–NSC) database. This database comprises 1 million individuals, selected by random sampling of 2% of the total 50 million people in the Republic of Korea. It serves as a representative sample cohort with evenly distributed sex, age, economic status, and residential areas. The database includes demographic data with diagnostic codes based on the International Classification of Diseases, 10th revision (ICD-10), as well as treatment history, patient visit dates, and medications.

### 2.1. Study population with septal deviation: SD group

The definition of the patient cohort for SD was based on the ICD-10 diagnostic code J342, diagnosed between 2002 and 2004. The definition of the target disease for tinnitus is ICD-10 code H931. The exclusion criteria were: (1) patients with records of both tinnitus and SD, where tinnitus was diagnosed prior to or simultaneously with the SD diagnosis; (2) patients under 20 years of age; (3) patients diagnosed with SD during the period 2005 to 2013.

The starting point for the SD group was the date of SD diagnosis, and the endpoint was the date of the first diagnosis of tinnitus if a record of tinnitus diagnosis existed, or December 31, 2013, if no record of tinnitus diagnosis existed.

### 2.2. Propensity score matching and selection of the control (non-SD) group

The control group was assembled using 1:2 propensity score (PS) matching, considering 7 independent variables (age, sex, residential area, economic status, history of hypertension, history of diabetes mellitus, and history of chronic kidney disease). PS matching was performed using a “greedy nearest neighbor” algorithm with a 1:2 ratio. The success of PS matching was confirmed by the absence of major imbalances in the standardized mean differences between the groups. All variables and diseases mentioned through PS matching were included in both the control group and the study group.

### 2.3. Outcome variables and statistical analysis

Two data analysts (CDY, SWY) independently conducted the data analysis between January and April 2021, with any discrepancies resolved through discussion. The unadjusted hazard ratio (HR) was calculated by considering only 2 variables, 1 independent and 1 dependent, without adjusting for other variables. The adjusted HR was obtained using the Cox Proportional-Hazards model, which accounted for all other variables. The risk ratio or relative risk was calculated as the ratio of the probability of an outcome in the SD group to the probability of an outcome in the non-SD group. The cumulative hazard ratio was obtained through Kaplan–Meier survival analysis, and the R 3.5.3 statistical program (R Foundation for Statistical Computing, Vienna, Austria) was used to analyze the results. The SAS program (SAS Institute Inc, Cary, NC) was also used for analyzing the NHIS–NSC database.

### 2.4. Subgroup analysis

In subgroup analyses, we examined sex, economic status, age, residential area, and underlying diseases within the SD group. We also evaluated septoplasty as a moderator variable.

### 2.5. Ethical considerations

All studies were conducted and designed in accordance with the Declaration of Helsinki using the NHIS–NSC database. The research was approved by the Institutional Review Board of Jeonbuk National University Hospital (IRB file number 2021-06-015). Informed consent was waived by the Institutional Review Board due to the retrospective nature of the study.

## 3. Results

This study followed a total of 32,370 individuals (10,790 in the SD group and 21,580 in the non-SD group) over a 9-year period, from January 2005 to December 2013. During this time, we examined the incidence of tinnitus and its relationship to septal deviation.

### 3.1. Validation of PS matching

**Figure 1.**
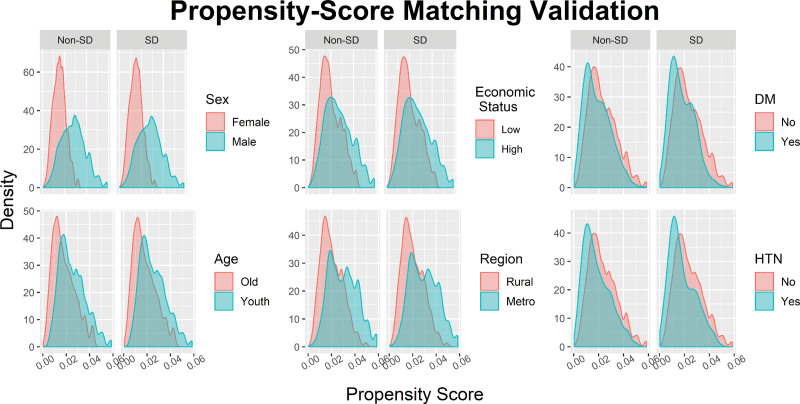
Validation of propensity score matching. HTN = hypertension; DM = diabetes mellitus.

PS matching was conducted to ensure comparability between the SD and non-SD groups. We matched the groups on key variables, including age, sex, residential area, economic status, and history of comorbid conditions such as hypertension, diabetes mellitus, and chronic kidney disease. After matching, the standardized mean differences between the groups were close to 0 for all variables except tinnitus and septoplasty, confirming the success of the matching process (Table 1). The similar distributions of the SD and non-SD groups (Fig. 1) further validated the effectiveness of the PS matching.

**Table 1:** Demography of SD and non-SD groups.

Variable	Control (non-SD) group (n = 21,580)	Study (SD) group (n = 10,790)	SMD
Sex			0.001
Male	13,574	6784	
Female	8006	4006	
Age			<0.001
Young < 50	15,626	7811	
Old ≥ 50	5954	2979	
Residential area			<0.001
Metropolitan	6706	3353	
Rural	14,874	7437	
Economic status			<0.001
Low < 70%	11,784	5891	
High ≥ 70%	9796	4899	
HTN			0.013
Yes	940	500	
No	20,640	10,290	
DM			0.023
Yes	634	277	
No	20,946	10,513	
CKD			0.02
Yes	123	79	
No	21,457	10,711	
Tinnitus			0.614
Yes	1627	1187	
No	19,953	9603	
Septoplasty			0.12
Yes	0	1713	
No	21,580	9077	

CKD = chronic kidney disease, DM = diabetes mellitus, HTN = hypertension, SD = nasal septal deviation, SMD = standardized mean difference.

### 3.2. Incidence rate and HR for tinnitus

**Figure 2.**
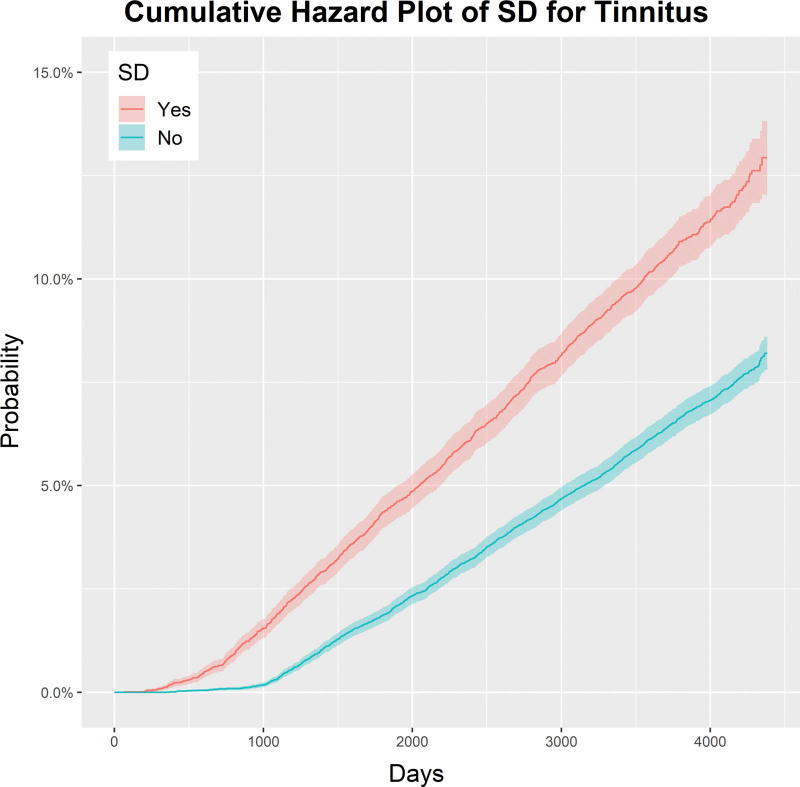
Overall cumulative hazard ratio for tinnitus in the SD group. SD = nasal septal deviation.

Table 2 shows the relationship between the 7 independent variables for tinnitus and 3 statistics: incidence rate (per 10,000-person years), unadjusted HR, and adjusted HR. The incidence rate is a measure of the frequency with which a disease occurs over a specified time period. Figure 2 and Table 2 show that the HR for tinnitus in the SD group was 1.74 (95% CI: 1.62–1.89), indicating that the prevalence of tinnitus was 1.74 times higher in the SD group compared with the control group.

**Table 2:** Incidence rate, adjusted and unadjusted HR for each group.

Variable	Study group total	Number of cases	Incidence per 100,000-person years	HR adjusted	HR unadjusted
Total	32,370	2814			
Group					
Control (non-SD)	21,580	1627	68.16	1	1
Study (SD)	10,790	1187	109.60	1.74 (1.62–1.89)	1.67 (1.55–1.8)
Septoplasty					
No	30,657	2675	81.18	1	1
Yes	1713	139	79.42	0.75 (0.63–0.9)	1 (0.84–1.18)
Sex					
Female	12,012	1231	95.28	1	1
Male	20,358	1583	72.67	0.73 (0.68–0.79)	0.76 (0.71–0.82)
Economic status					
Low < 70%	17,675	1415	74.61	1	1
High ≥ 70%	14,695	1399	88.90	1.12 (1.04–1.21)	1.19 (1.11–1.28)
Residential area					
Rural	22,311	2032	85.01	1	1
Metropolitan	10,059	782	72.41	0.81 (0.75–0.89)	0.85 (0.78–0.92)
Age					
Old ≥ 50	8933	1276	135.73	1	1
Young < 50	23,437	1538	60.79	0.45 (0.42–0.49)	0.45 (0.41–0.48)
HTN					
No	30,930	2596	78.26	1	1
Yes	1440	218	142.56	1.20 (1.04–1.39)	1.82 (1.58–2.09)
DM					
No	31,459	2701	80.11	1	1
Yes	911	113	114.42	0.95 (0.79–1.16)	1.42 (1.18–1.71)
CKD					
No	32,191	2791	80.93	1	1
Yes	179	23	106.87	1.01 (0.67–1.53)	1.32 (0.87–1.98)

CKD = chronic kidney disease, DM = diabetes mellitus, HR = hazard ratio, HTN = hypertension, SD = nasal septal deviation.

### 3.3. Subgroup analysis

**Figure 3.**
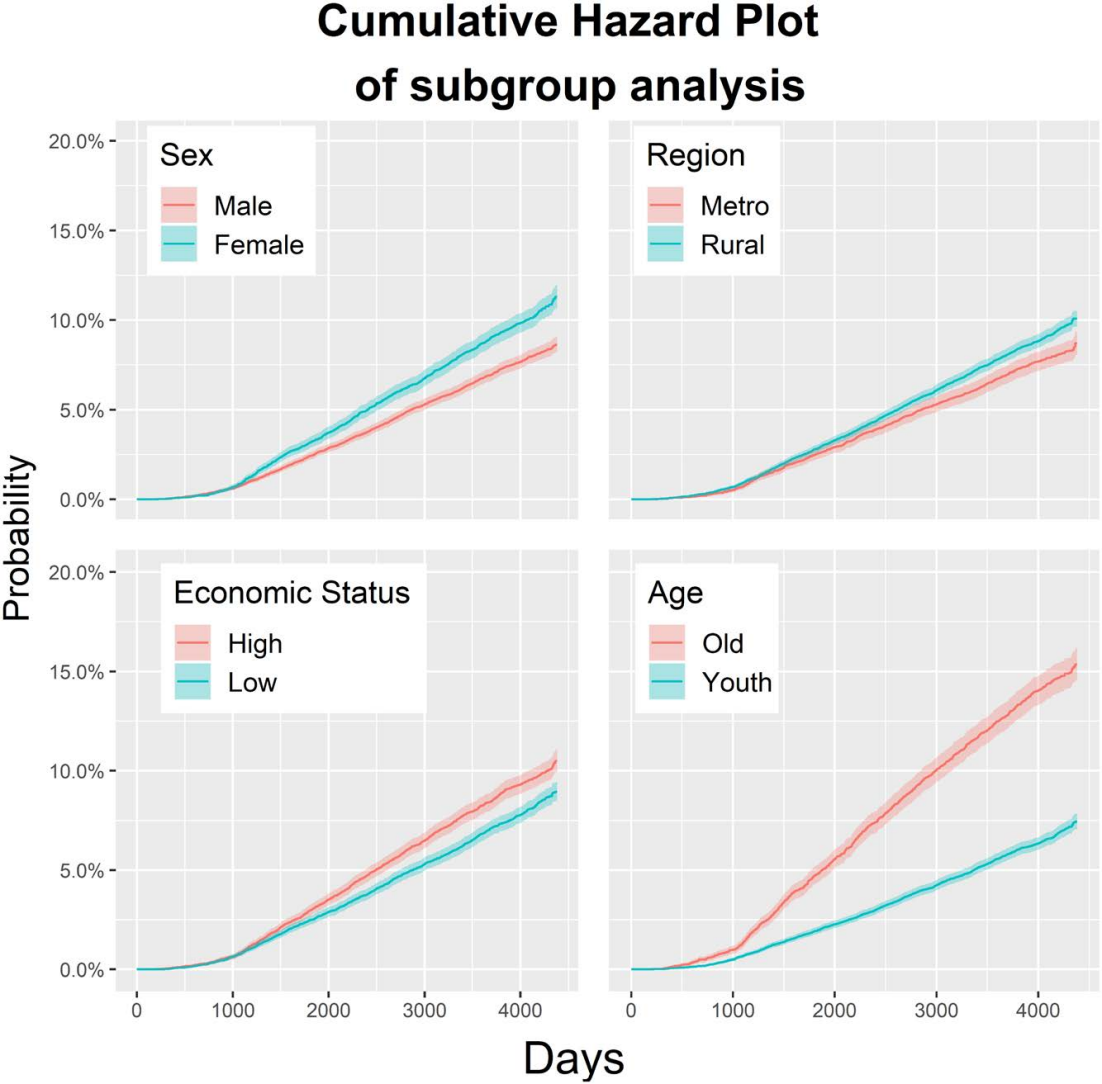
Cumulative hazard ratio plot for subgroups: sex, residential area, economic status and age.

In the subgroup analysis of demographic factors, the HR was 0.73 (95% CI: 0.68–0.79) in male patients compared with female patients. It was 1.12 (95% CI: 1.04–1.21) in patients with high economic status compared with those with low economic status, and 0.81 (95% CI: 0.75–0.89) in patients living in cities compared with those living in rural areas. Additionally, HR was relatively low at 0.45 (95% CI: 0.42–0.49) in younger patients (<50 years) compared with older patients (Fig. 3).

**Figure 4.**
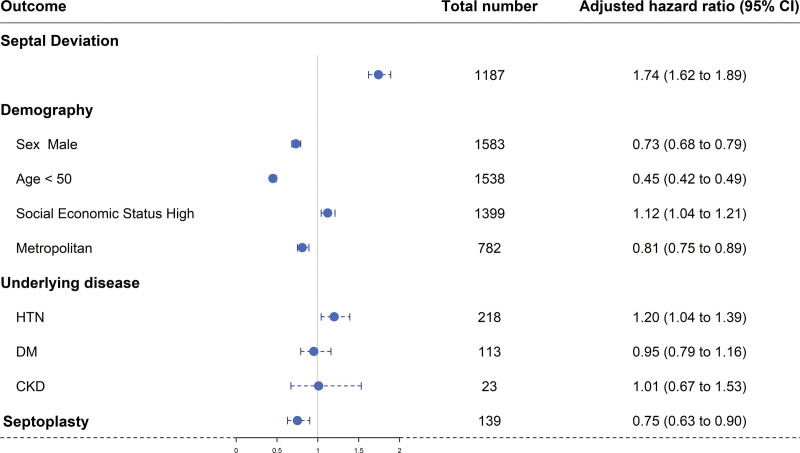
Forest plot of cumulative hazard ratio (mean with 95% confidence interval) for each factor. SES = socioeconomic status; RA = residential area; CKD = chronic kidney disease.

Figure 4 is a forest plot showing the HRs of each variable. In the subgroups excluding diabetes mellitus, there was a statistically significant difference in HRs between the subgroups.

Among the 10,790 patients in the SD group, 1713 underwent septoplasty. The HR for developing tinnitus after septoplasty was 0.75 (95% CI: 0.63–0.90) compared to those who did not undergo surgery, indicating that patients who received septoplasty had a 25% lower risk of developing tinnitus. This suggests that correcting septal deviation may alleviate Eustachian tube dysfunction and other middle ear pathologies, which could reduce the risk of tinnitus development.

## 4. Discussion

SD is closely associated with obstructive sleep apnea (OSA) and sleep disorders, which are believed to contribute to tinnitus. According to Silvoniemi et al, OSA, which can occur during sleep due to SD, may lead to sleep disorders.^[9]^ Several studies have shown that sleep disorders are risk factors for depression, anxiety, and tinnitus.^[10–13]^ Therefore, SD, which is closely related to sleep disorders, including OSA, may be associated with tinnitus. Our study demonstrated that SD increases the risk of tinnitus and that SD correction (septoplasty) reduces the risk of tinnitus development.

Mechanistically, SD may contribute to the development of tinnitus through several pathways. First, SD can lead to chronic nasal obstruction, which may result in increased negative pressure in the middle ear due to impaired Eustachian tube function. This negative pressure can cause Eustachian tube dysfunction, leading to an imbalance in middle ear pressure, which may manifest as tinnitus.^[14]^ Second, chronic nasal obstruction caused by SD can lead to hypoxia during sleep, particularly in patients with concurrent OSA. Hypoxia has been shown to affect the auditory pathways, potentially increasing the susceptibility to tinnitus.^[15]^ Furthermore, SD can cause chronic mouth breathing, which may exacerbate orofacial tension and muscle strain, particularly in the temporomandibular joint area. Temporomandibular joint disorders are known to be associated with tinnitus, and the altered biomechanics of the jaw and ear due to chronic mouth breathing may contribute to tinnitus development in SD patients.^[16]^ Finally, the chronic stress and discomfort caused by SD-related symptoms, including nasal obstruction and poor sleep quality, can lead to heightened central nervous system sensitivity, which is a recognized risk factor for tinnitus.^[17]^

In patients with hypertension, tinnitus may arise from vascular changes leading to reduced blood flow to the cochlea, causing auditory system ischemia.^[18]^ Endothelial dysfunction and oxidative stress associated with hypertension can further aggravate tinnitus, while blood pressure fluctuations may disrupt cochlear fluid balance, contributing to tinnitus development.^[19,20]^

The association between tinnitus and socioeconomic status has been reported in previous population-level or case-control studies.^[21–23]^ These studies showed that individuals with lower socioeconomic status are at an increased risk of developing tinnitus. However, in this study, higher socioeconomic status was identified as a risk factor for tinnitus. This discrepancy may have arisen because this study did not account for other socioeconomic factors such as occupation, education level, and marital status. It is also possible that individuals with higher socioeconomic status are more likely to visit hospitals and be diagnosed with SD and tinnitus, leading to an economic bias. Further research is needed to account for other socioeconomic factors, such as occupation, in future studies.

Xu et al found that tinnitus is more prevalent in people living in rural areas compared with urban areas.^[24]^ The present study also found a lower risk of tinnitus in patients with SD residing in urban areas. This may be because urban areas are noisier, potentially masking the perception of tinnitus.

Earlier studies have shown that female sex and older age are risk factors for tinnitus.^[1,21,25]^ Similarly, this study found that tinnitus occurs more frequently in female and older individuals. The HR for developing tinnitus was lower in younger (<50 years) and male SD patients compared with older and female patients. Identified as significant risk factors. Older age is strongly associated with an increased prevalence of tinnitus, which is likely due to the natural deterioration of auditory pathways and the cumulative effect of hearing loss over time. Several studies have confirmed that individuals aged over 55 years are at a considerably higher risk of developing tinnitus compared to younger populations. This is likely due to age-related auditory system degeneration and other age-related changes, such as vascular changes affecting cochlear function. Moreover, female sex has also been recognized as a risk factor for tinnitus. Women are not only more likely to develop tinnitus, but they also frequently report higher levels of tinnitus-related distress, including sleep disturbances, hearing difficulties, and comorbid conditions such as hypertension and anxiety. In particular, older women are disproportionately affected by bothersome tinnitus, which significantly impacts their quality of life. These findings are supported by data from various population-based studies, which show that females, especially those over 50 years, report tinnitus more often and with greater severity compared to their male counterparts. Previous studies have shown that anxiety disorders, depression, ischemic stroke, ischemic heart disease, hypertension, and diabetes are related to tinnitus.^[26]^ However, this study did not find a higher risk of tinnitus in SD patients with hypertension or diabetes. Further studies are required to explore the impact of other underlying diseases known to be risk factors for tinnitus.

To the best of our knowledge, this is the first study to use large-scale real-world data to evaluate the risk of tinnitus in individuals with nasal septal deviation. Although we have demonstrated significant findings, several limitations should be considered. First, SD and tinnitus were only identified by diagnostic codes, without detailed information such as patient symptoms or physical examination findings. However, we attempted to improve the diagnostic accuracy of SD and tinnitus by including patients who underwent computed tomography and pure-tone audiometry. Second, this study did not able to confirm whether individuals experienced concurrent symptoms of chronic rhinosinusitis, such as nasal congestion, facial pain, or loss of smell, which may have influenced both SD and tinnitus outcomes. Third, this study did not consider factors such as family history, lifestyle, or stressful life events. Further research that comprehensively examines these factors is necessary to confirm the effect of SD on tinnitus.

## 5. Conclusion

This observational study found that SD was associated with a higher rate of tinnitus. According to the results of this study, older age, female sex, high socioeconomic status and rural residential area increased the risk of developing tinnitus. Therefore, it is important to be aware that tinnitus may occur more frequently in patients with SD, and to pay attention to treating their tinnitus so that it does not affect the patients’ quality of life.

## Author contributions

**Conceptualization:** Cha Dong Yeo, Jong Seung Kim.

**Data curation:** Sang Woo Yeom, Jong Seung Kim.

**Formal analysis:** Cha Dong Yeo, Sang Woo Yeom.

**Methodology:** Sang Woo Yeom, Jong Seung Kim.

**Supervision:** Eun Jung Lee.

**Validation:** Sang Woo Yeom, Eun Jung Lee.

**Writing – original draft:** Cha Dong Yeo, Jong Seung Kim.

**Writing – review & editing:** Cha Dong Yeo, Eun Jung Lee, Jong Seung Kim.
